# Women at heart: Introducing gender cardio-oncology

**DOI:** 10.3389/fcvm.2022.974123

**Published:** 2022-11-23

**Authors:** Maria Laura Canale, Irma Bisceglia, Giuseppina Gallucci, Giulia Russo, Andrea Camerini, Stefania Angela Di Fusco, Andrea Paccone, Massimiliano Camilli, Damiana Fiscella, Chiara Lestuzzi, Fabio Maria Turazza, Michele Massimo Gulizia, Daniela Pavan, Nicola Maurea, Domenico Gabrielli, Fabrizio Oliva, Furio Colivicchi

**Affiliations:** ^1^Division of Cardiology, Azienda USL Toscana Nord-Ovest, Versilia Hospital, Lido di Camaiore, Italy; ^2^Integrated Cardiology Services, Department of Cardio-Thoracic-Vascular, Azienda Ospedaliera San Camillo Forlanini, Rome, Italy; ^3^Cardio-Oncology Unit, IRCCS-CROB, Rionero in Vulture, Italy; ^4^Department of Cardiovascular and Sports Medicine, Azienda Sanitaria Universitaria Giuliano Isontina (ASUGI), Trieste, Italy; ^5^Department of Medical Oncology, Azienda USL Toscana Nord-Ovest, Versilia Hospital, Lido di Camaiore, Italy; ^6^Department of Clinical and Rehabilitation Cardiology, Ospedale San Filippo Neri, Rome, Italy; ^7^Department of Cardiology, G. Pascale National Cancer Institute Foundation (IRCCS), Naples, Italy; ^8^Dipartimento di Scienze Cardiovascolari e Pneumologiche, Università Cattolica del Sacro Cuore, Rome, Italy; ^9^Dipartimento di Medicina Cardiovascolare, Fondazione Policlinico Universitario A. Gemelli IRCCS, Rome, Italy; ^10^U.O.C. Cardiologia, Ospedale Garibaldi-Nesima, Azienda di Rilievo Nazionale e Alta Specializzazione “Garibaldi”, Catania, Italy; ^11^Cardiology Unit, Department of Oncology, CRO National Cancer Institute, Aviano, Italy; ^12^Cardiology Unit, Fondazione IRCCS Istituto Nazionale dei Tumori, Milan, Italy; ^13^S.C. Cardiologia Pordenone, Azienda Sanitaria Friuli Occidentale, Pordenone, Italy; ^14^Division of Cardiology, Azienda Ospedaliera San Camillo-Forlanini, Rome, Italy; ^15^Cardiologia 1- Emodinamica Dipartimento Cardiotoracovascolare “A. De Gasperis”, ASST Grande Ospedale Metropolitano Niguarda, Milan, Italy

**Keywords:** cardio-oncology, gender medicine, radiotherapy, anthracyclines, immunotherapy

## Abstract

As cardio-oncology imposed itself as the reference specialty for a comprehensive cardiovascular approach to all patients with cancer, a more specific and careful cardiac evaluation of women entering their journey into cancer care is needed. Gender medicine refers to the study of how sex-based biological and gender-based socioeconomic and cultural differences influence people’s health. Gender-related aspects could account for differences in the development, progression, and clinical signs of diseases as well as in the treatment of adverse events. Gender also accounts for major differences in access to healthcare. As for medicine and healthcare in general, gender-related characteristics have gained significance in cardio-oncology and should no longer be neglected in both clinical practice and research. We aimed to review the most relevant cardiovascular issues in women related to the cardio-oncology approach to offer a specific gender-related point of view for clinicians involved in the care process for both cancer and cardiovascular disease.

## Introduction: The need for gender cardio-oncology

Cardio-oncology (CO) now leads the cardiology care pathway for patients with cancer and provides guidance for clinicians involved in this challenging management. European and American cardiology and medical oncology scientific societies released guidelines and recommendations (1–3) on CO and an increasing number of national cardiology societies have published CO reports (4–6). Bearing in mind the well-established role of CO in clinical practice, a step ahead toward a more focused CO approach on women entering their journey into cancer care is needed.

In truth, there are differences between men and women in the frequency, symptomatology, and severity of many diseases, as well as in the responsiveness to therapies and adverse drug responses (7, 8). In clinical practice, a sex-based approach promotes the appropriateness and personalization of care with the goal to improve quality of life (9). It advocates for a new approach to medicine, recommending policies targeted at establishing new preventive, diagnostic, prognostic, and therapeutic health measures that take gender variations into consideration. Biological and clinical parameters, as well as cultural and socio-psychological factors, should all be taken into account. Despite the fact that there are known biochemical and sex-related factors that influence the risk of disease in women, the connections between various diseases in women are still understudied (10). Understanding the temporal pattern of the illness network may assist promote a life-course approach to women’s health and uncover crucial indicators to decrease the risk of future bad outcomes, which is critical for providing cost-effective and improved healthcare for women (7).

This CO sex and gender-oriented paradigm shift will try to fill the gap in offering a more tailored clinical approach.

**FIGURE 1:**
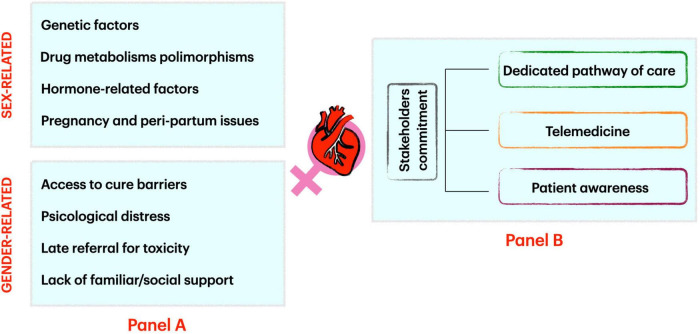
Factors influencing sex and gender-related cardio-oncology issues **(Panel A)** and how to address them **(Panel B)**.

We analyzed the most important cardiovascular issues in women related to CO approaches to provide a gender-specific perspective for doctors working in cancer and cardiovascular disease care (Figure 1, panel A).

## Sex differences in heart failure

Heart failure (HF) is a complex syndrome characterized by structural and functional impairment of left ventricle. It can be considered a significant public health issue, as its prevalence is rising (about 1–2% of adults in western countries) with high morbidity and mortality rates (11). Important sex differences are represented in HF: Etiology, clinical characteristic, and prognosis are different between men and women. Of note, women are underrepresented in HF clinical trials (12). Prevalence data show no difference between men and women; however, women are more likely to be affected by heart failure with preserved ejection fraction (HFpEF), while heart failure with reduced ejection fraction (HFrEF), where ischemic component is predominant, is more represented in men (13–15). Peripartum cardiomyopathy and certain genetic X-linked cardiomyopathies such as Duchenne or Becker dystrophies are special clinical HF scenarios of women (16) as is chemotherapy-related cardiomyopathy due to anthracycline or Her-2 therapy cardiotoxicity in breast cancer (17). Takotsubo syndrome is predominant in women; its etiopathology is not completely clear, but it seems that a decrease in estrogen levels during the menopausal period could increase the sensitivity of the heart in catecholamine circulation and be responsible for this clinical manifestation (18).

Traditional cardiovascular risk factors (CVRFs) have a different impact in male and female risk of developing HF. It is quite difficult to generalize as the prevalence of traditional CVRF differs greatly around the world, but the impact of cigarette smoking, diabetes, hypertension, and obesity in causing HF seems to be greater in women (19–24). Women have also sex-specific risk factors: Gestational diabetes and hypertension are predisposing conditions to develop HF in the following years (25–27).

## Anthracycline cardiotoxicity in women

Anthracyclines represent the cornerstone for the treatment of many solid and hematological cancers; their cardiac toxicity is known from decades and ranges from asymptomatic reduction of left ventricle ejection fraction (LVEF) to symptomatic heart failure (28). Several treatment and patient-related items are described as risk factors for anthracycline cardiotoxicity. Among those patient-related, female sex and age deserve special consideration. Moreover, there is a significant clinical difference between pediatric and adult doxorubicin-induced cardiotoxicity.

For young girls who survived cancer in pediatric age, cardiotoxicity risk is approximately four times greater than the risk for male childhood cancer survivors treated with anthracyclines (29). Lipshultz et al. reported that left ventricular contractility of female childhood cancer survivors 8 years after completing doxorubicin treatment was significantly worse than that of their male counterparts and the female sex was an independent factor for cardiac dysfunction (30). In Mulrooney’s study, the relative hazard of congestive heart failure was 40% higher in female survivors than in male survivors after childhood cancer (31). Of note, not all clinical studies or registries on cancer survival identify the female sex as a risk factor for cardiotoxicity. In a large cohort of Danish childhood cancer survivors, no evidence of the female sex as a risk factor for HF was found (32, 33).

On the contrary, studies that analyze sex-related differences in cardiac side effects in adult cancer population showed that the male sex has an increased risk for cardiovascular events and these differences could be explained (at least in part) by the presence of cardiac pre-existent disease, although post-menopausal women seem to be as susceptible to cardiotoxicity as men. In a population-based cohort study of chemotherapy-treated Hodgkin lymphoma with pre-existing cardiac heart disease, the male sex was a significant risk factor (34).

Another issue to explain this difference is due to the fact that most of the research on anthracycline cardiotoxicity in an adult cancer population are conducted in breast cancer, that is, primarily a female-related cancer. Similarly, in another large cohort study, patients with cancer who developed cardiac events (heart failure and cardiac death) were significantly older, predominantly men with pre-existing cardiac risk factors and history (35).

Very few pre-clinical studies with juvenile animal models can help to understand the sex difference in anthracycline cardiotoxicity. In pre-clinical studies, with adult animal models, the female sex is protective against anthracycline cardiotoxicity compared to the male sex both in the acute and chronic setting (36, 37).

Reasons to clearly explain sex differences in cardiac toxicity from anthracyclines are lacking. Some hypotheses have been proposed as the role of female hormones in oxidative stress and in mitochondrial dysfunction (both pathways are believed to be involved in the genesis of cardiac damage from doxorubicin) (38–41). Last, the role of pharmacokinetics differences between male patient and female patient cannot be excluded (42).

It could be concluded that the female sex is a risk factor for anthracycline cardiotoxicity in patients with childhood cancer, while it seems to be protective in adult fertile women. Post-menopausal patients with cancer have the same cardiac risk of the elderly men.

## Cardiac issues of cancer treatment during pregnancy

Cancer diagnosis during pregnancy should be considered as a rare situation in oncology with an estimated incidence of one case every 1000 pregnancies (43). An increase in incidence in the next decades can be expected, in particular in Western countries, due to an older age at first pregnancy (44) and to the wider use of non-invasive prenatal testing that may identify early-stage non-symptomatic malignancies (45). Breast cancer represents the most common cancer type found during pregnancy, but the incidence of other types (as cervical cancer, lymphoma, ovarian cancer, and leukemia) is not negligible (46). When a diagnosis of pregnancy-associated cancer is made, the patient should be referred to a center with specific expertise and managed by a multidisciplinary expert team (47). Breast surgery is feasible throughout the pregnancy, while radiotherapy should be postponed until after delivery due to the high risk of fetal abnormalities (48, 49).

The use of chemotherapy during pregnancy should be avoided during the first trimester due to the high risk of fetal malformations, but it is considered safe during the second and third trimesters. In the first 12 weeks of pregnancy, the placenta does not effectively protect the fetus against the effects of cytotoxic drugs, so anticancer agents could interfere with organogenesis leading to an increased risk of miscarriage and congenital malformations (50, 51). After the first trimester, chemotherapy can be safely administered because the incidence of fetal malformations overlaps with that of the general population (52). Anthracyclines, cyclophosphamide, and taxane-based regimens are widely used for the treatment of patients with breast cancer (53, 54). The cardiotoxic effects of anthracyclines during pregnancy (second and third trimester) on women do not differ from those of the general cancer population, and the same precautionary rules to reduce the risk of cardiac side effects should be followed (2). Although chemotherapy is generally considered safe after the first 12 weeks of pregnancy, an increased risk of prematurity and rupture of membranes was reported in a large population study on 11 milion births (55); hence, caution is required. To explain this effect, a direct anthracycline-related vascular damage of placenta has been proposed. Doxorubicin-exposed pregnant mice showed a vascular-derived placental toxicity with a reduced blood flow and a lower birth weight (56).

Population data focused on the long-term cardiac and general safety outcome of children with *in utero* exposure to chemotherapy. Overall, retrospective cohort studies are reassuring with no evidence of after-birth cardiac issues compared to babies born from healthy women (57). A prospective case–control study compared 129 children with *in utero* exposure to anticancer agents in the second or third trimester with 129 matched control children without exposure. The authors did not report any clear adverse effects on growth, cognitive, and cardiac function in early childhood even if the incidence of preterm birth and small gestational age was higher among the exposed group (48).

The second drug group historically related to cardiac toxicity is anti-HER2 agents. The use of trastuzumab during pregnancy is contraindicated in relation to the increased risk of developing oligo- and/or anhydramnios. A meta-analysis on 30 patients recently reported a total of 32 fetuses in trastuzumab-exposed women mainly in the metastatic setting. Oligohydramnios or anhydramnios was the most common (58.1%) adverse event reported. There was a statistically significant decrease in its incidence in patients receiving trastuzumab only during the first trimester. In 43.3% of cases, a completely healthy neonate was born. About 41.7% of fetuses exposed to trastuzumab during the second and/or third trimester were born completely healthy versus 75.0% of fetuses exposed exclusively in the first trimester (58). Few data are available for newer anti-HER2 agents. A recent report focused on pregnancy issues in ALTTO and NeoALTTO trials, both testing trastuzumab and lapatinib in patients with early breast cancer. Despite both protocols, as usual, required active contraception for women with childbearing potential, 12 women exposed to anti-HER2 therapy or immediately after treatment completion became pregnant. Seven patients opted for an induced abortion, while five completed the pregnancy. All pregnancies and deliveries had no complications, and no congenital anomalies were reported (59). Given the strong recommendation against the use of anti-HER2 agents, no data are available for other anti-HER2 agents such as pertuzumab, trastuzumab emtansine, and neratinib, and thus their administration in pregnant women is contraindicated.

## Cardiovascular adverse events during pregnancy after exposure to cardiotoxic therapies in survivors of childhood, adolescent, and young adult cancers

Improvements in anticancer global strategy resulted in better outcomes for a large number of patients with cancer, with many of them experiencing definitive cure or long-term survival. In particular, the survival rate for childhood, adolescent, and young adult (CAYA) cancers peaked near 85% with a consequent steadily growing population of long-term survivors (60). As a consequence, more than 1,000,000 survivors of CAYA cancer can be identified across North America and Europe (61, 62). Survivors of CAYA cancers are at risk for late toxicities from anticancer therapies as well as psychological and social issues, and an increased incidence of comorbidities has been reported (63–65). Late cardiovascular sequelae are a major concern for this group of patients and are mainly related to chest radiation therapy and anthracycline exposure (66, 67). On these grounds, it is not surprising that cardiovascular safety of pregnant women previously exposed to cardiotoxic anticancer treatments requires special attention.

Hines and colleagues described the outcome of 1554 pregnancies among 847 female cancer survivors. They reported an overall very low incidence rate of cardiomyopathy during pregnancy (0.3%), slightly increased taking into account postpartum and pre-pregnancy cardiomyopathy. The only risk factor for pregnancy-related cardiomyopathy was a higher median dose of anthracyclines received (68). As a consequence, the authors stated the general cardiac safety of pregnancy in CAYA cancer survivors but highlighted the need for a careful evaluation and follow-up during pregnancy (and later on) in women with a history of anthracycline exposure and/or a documented previous or current subclinical or symptomatic cardiomyopathy. Similar results have been reported in a Canadian series of 78 women (94 pregnancies) treated with cancer therapy as CAYAs. The majority of cases received anthracyclines, while around one-third received non-anthracycline-based chemotherapy and/or radiation therapy. The observed risk of developing heart failure during pregnancy was very low in female CAYA cancer survivors without a history of cardiotoxicity, while those with a history of cardiotoxicity have approximately 30% chance of developing heart failure and so should be offered a close cardiac monitoring program by an expert multidisciplinary team (69). A previous report on a small population of female survivors of childhood cancers pointed out the safety of pregnancy from a cardiac point of view but, once more, those women presenting with left ventricular dysfunction before pregnancy were at risk for worse outcome during and after pregnancy (70). M.D. Anderson Cancer Center Experience on this topic has been reported few years ago. Compared to a matched control group of female survivors of CAYA cancers, pregnancy represented a risk factor for adverse cardiac events as well as a higher anthracyclines cumulative dose and a longer time from cancer treatment to first pregnancy (71). Van Dalen et al. reviewed 53 childhood cancer survivors with a total of 100 deliveries. Two of these patients had a history of acute congestive heart failure related to anthracyclines therapy. No heart failure event occurred during pregnancy leading to a 0% incidence rate but, as the authors stated, larger cohort studies with adequate power and long-term follow-up are needed (72). A recently published retrospective analysis on 64 women and 110 pregnancies reported a slightly higher incidence of cardiac events in female CAYA cancer survivors. A total of five women (7.8%) had peripartum cardiac events (symptomatic and subclinical). Symptomatic dysfunction without prior cardiac dysfunction incidence was lower (1.8%), but represented a 55-fold increased risk compared to the general population. Risk factors were younger age at cancer diagnosis and higher anthracyclines dose. Of note, in a total of five cases, cardiac function recovery after delivery occurred in one case only (73) (Table 1).

**TABLE 1:** Summary of published reports on cardiac outcome during and after pregnancy in survivor women of childhood, adolescent, and young adult cancers.

Authors	Year	Type	Population/Pregnancies	Cardiac outcome
Hines et al. (68)	2016	Retrospective	847/1554	Overall very low incidence (0.3%) of cardiomyopathy but warning in case of previous cardiac toxicity, Anthra exposure or documented cardiomyopathy
Liu et al. (69)	2018	Retrospective	78/94	Low incidence (5.3%) of heart failure in general population. All cases occurred in women with a history of cardiotoxicity
Bar et al. (70)	2003	Prospective	37/72	Overall favorable outcome but warning in those patients with left ventricle disfunction before pregnancy
Thompson et al. (71)	2017	Retrospective	337/86	Increased incidence of adverse cardiac events in pregnant vs. non-pregnant survivors. Higher Anthra cumulative dose and longer time to first pregnancy were risk factors for adverse cardiac events.
van Dalen et al. (72)	2006	Retrospective	53/100	No heart failure event reported
Chait-Rubinek et al. (73)	2019	Retrospective	64/110	Peripartum cardiac events were uncommon but incidence was not negligible. Younger age at cancer diagnosis and a higher Anthra cumulative dose were risk factors.

Anthra = anthracyclines.

A recent meta-analysis of six studies consisting of 2,016 pregnancies, predominantly in childhood cancer survivors, clearly highlighted the very low rate of pregnancy-related cardiac events in the general population. Only 33 cardiac events were reported leading to an overall weighted incidence of left ventricular dysfunction or heart failure of 1.7%. A sharp increase in incidence was noticed in patients with a history of cardiac toxicity from previous anticancer therapy. While the incidence of cardiac adverse events was 0.24% in cases without previous cardiac toxicity, it peaked to 28.4% in women with a history of cardiac side effects translating into an odds ratio of 47.4 for the increase in the risk of heart failure and left ventricular dysfunction (74). A population-based cohort analysis on obstetrical and perinatal outcomes in CAYA cancer survivors showed that female survivors had an increased risk for maternal cardiac morbidity (75).

## Sex influence in radiation-associated cardiac disease

Unintended irradiation of healthy tissues surrounding tumor can elicit endothelial dysfunction that leads to inflammatory responses and subsequent vascular damage (76, 77). These phenomena cause the so-called radiation-associated cardiac disease (RACD), an umbrella term that encompasses myocardial fibrosis with a possible evolution in myocardial dysfunction and congestive heart failure, pericarditis, valvular heart disease, conduction abnormalities, and vascular disease including coronary artery disease (CAD). The vascular damage can occur in the carotid and intracranial arteries when head and neck tumors are irradiated, in the coronary arteries when lymphomas, breast, lung, esophageal, and gastric cancers are irradiated, and in the aorta, renal, intestinal, and peripheral arteries in lymphoma, intestinal, and testicular cancers (78, 79). The hallmark of radiotherapy(RT)-induced vascular damage is media disruption, fibrosis and atrophy, and adventitial thickening and fibrosis; intimal plaques are not different from those observed in non-irradiated patients, with a fibrocalcific component more prominent than a proliferative component (80, 81). Patients surviving for many decades after treatment showed late cardiotoxic effects of the radiation therapy, mostly CAD events. Modern techniques have banned extended fields and have modified delivery techniques to reduce cardiac exposure, but a mean heart dose > 10Gy can still be needed and can significantly increase cardiovascular disease mortality risk (82).

Coronary artery disease is the most frequent cardiotoxic phenotype after thoracic RT, and this is the point where sex becomes an issue. We know that women have different clinical presentations of CAD if compared with men and that genetic, anatomic, physiologic, psychosocial, cultural, and economic factors account for the different clinical phenotypes. CAD in male patients affects mainly epicardial coronary arteries, whereas in female patients the microvascular circulation has the greatest impact. These differences will be translated in the CAD phenotype of RACD (83–86). In female patients, traditional cardiovascular risk factors such as tobacco use, obesity, type 2 diabetes mellitus, depression, and psychosocial stress have a more powerful impact on CVD compared to male patients (87). In more than 2,000 female patients treated with RT for breast cancer from 1958 to 2001, baseline risk factors accounted for a 2-fold increased risk of major cardiovascular events and a history of CAD for a 6-fold increased risk (88). Sex-related differences in RACD can be studied mostly in patients with lymphoma and in patients with pulmonary malignancies. A reliable comparison of cardiotoxicity between male patients and female patients cannot be done in breast cancer, a malignancy studied almost exclusively in the female gender.

A recent pre-clinical study investigated the molecular basis of sex-specific differences in toxicity following localized radiotherapy in male and female mice exposed to 19Gy cardiac irradiation; female mice showed increased tolerance to radiotherapy, and this cardio-protective effect was proven to be dependent on estrogens via a Rho-B-activated estrogen pathway (89). Unfortunately, in the clinical setting, very few studies have made a comparison of RACD in male patients and female patients. In a study performed with old radiotherapeutic techniques (between 1969 and 1998), 1279 patients with clinical Stage IA-IVB Hodgkin lymphoma were treated with mediastinal RT and followed up for a median time of almost 15 years; in these patients, old age and male sex predicted the occurrence of cardiac events and this fact was supposed to be linked to a higher proportion of cardiovascular risk factors in male patients (90). In a more recent review of 10 studies (four prospective and six retrospective), with a population of 13,975 patients (41% female patients and 59% male patients), a 4-fold increased rate of cardiovascular events and a 2-fold increase on all-cause mortality in women were observed following radiation therapy for Hodgkin lymphoma (91). Moreover, even though both male patients and female patients had higher mortality rates with advancing age, this effect was higher in female patients. The reason for this disadvantage of female patients in RACD has not received a full explanation. It could be due to the reduced presence of women in these clinical trials, to the higher doses of radiation needed to treat Hodgkin lymphoma in women, and to the more frequent microvascular phenotype of CAD in women. Overall the higher risk of radiation therapy is independent from cardiovascular traditional risk factors (88). There are also female-specific risk factors associated with an increased risk of cardiovascular issues that need to be addressed when evaluating global cardiovascular risk of women in which a thoracic radiotherapy is planned, especially young and middle-aged women in the adjuvant setting: a history of adverse pregnancy outcomes (e.g., preeclampsia and gestational hypertension, gestational diabetes, and preterm delivery), early-onset menopause, polycystic ovarian syndrome, breast or ovarian cancer, and inflammatory disorders such as rheumatoid arthritis, psoriasis, and systemic erythematous lupus. When chest radiotherapy is planned for patients with pre-existing traditional and/or female-specific risk factors, a tailored pre-treatment evaluation, an aggressive treatment of risk factors, and a personalized monitoring are mandatory. Even though a sex specificity for adjuvant RT in breast cancer cannot be assessed, it is important to be aware of the importance of a careful history in female patients with breast cancer. Table 2 summarizes most significant published evidence on RACD.

**TABLE 2:** Summary of published evidence on cardiac toxicity of radiotherapy.

Authors	Year	Type	Population	Cardiac outcome
Darby et al. (88)	2013	Population-based case-control study. Follow-up 0–20 years	2168 women treated with RT for breast cancer in the years between 1958 and 2001 in Sweden and Denmark. Estimation of the mean radiation dose to the whole heart (MHD) and to the left anterior descending artery was performed.	963 major coronary events were documented; the incidence of major coronary events started within 5 years after RT, increased linearly with the mean dose to the heart and continued for at least two decades. A greater absolute risk was observed in those with pre-existing CVRF.
Galper et al. (90)	2011	Retrospective Median follow-up: 14.7 years	1279 Hodgkin lymphoma patients treated with mediastinal irradiation between 1969 and 1998 in Harvard-affiliated hospitals.	636 cardiac events in 187 patients, cardiac procedures in 89 patients. Absolute excess risk of irradiated patients was 18.2 for CABG, 19.3 for PCI, 9.4 for implantation of an ICD or a PM, 14.1 for pericardial surgery. Older age at diagnosis and male gender predicted cardiac events.
Khalid et al. (91)	2020	systematic review and network meta-analysis of 10 studies (4 prospective, 6 retrospective).	13,975 Hodgkin’s Lymphoma patients (41% females, 59% males)	CV events/mortality significantly higher in women compared to men. All-cause mortality was also higher in women compared to men. Elderly populations showed a higher rate of mortality, which was even higher for women than men
Van Nimwegen et al. (93)	2015	case-control study	2617 five-year survivors of Hodgkin lymphoma diagnosed before age 51 years and treated with radiotherapy and/or chemotherapy between 1965 and 1995. Estimation of MHD and MLVD was performed.	91 cases of moderate to severe HF. HF rates increased at MHD greater than 25 Gy or MLVD greater than 15 Gy. Anthracycline-containing chemotherapy induced an almost 3-fold increase in HF rate.

RT, radiotherapy; CVRF, cardiovascular risk factors; CABG, coronary artery by-pass graft; PCI, percutaneous coronary intervention; ICD, implantable cardioverter defibrillator; PM, pacemaker; HF, heart failure; MHD, median heart dose; MLVD, median left ventricle dose.

As far as survivors of childhood malignancies are concerned, the female sex is considered a risk factor for cardiotoxicity, but the impact of RT alone has not been investigated (92). Concomitant chemotherapy (especially if anthracycline-based) increases the risk of cardiovascular disease (93). Other manifestations of RACD such as valvular heart disease, pericarditis, and conduction abnormalities are well-known and diffusely described, but there is no clear evidence of a sex effect.

## Sex-related differences of cardiac toxicity of immunotherapy

Immunotherapies have revolutionized the treatment of a variety of solid and hematologic cancers, but they come with their own set of side effects that vary depending on the kind of immunotherapy and are linked to the mechanism of action (94). Disinhibition of T-cell function by immune checkpoint inhibitors (ICIs) can lead to a spectrum of inflammatory side effects, or immune-related adverse events (irAEs). Although the specific pathophysiology of irAEs is unknown, multiple pathways have been hypothesized to account for their formation (95).

Sex-related differences in toxicity of ICIs have been described. Women treated with anti-programmed cell death protein 1 treatment are more likely to experience irAEs than male patients. In addition, specific irAEs, such as endocrinopathies and pneumonitis, were more common in women (96), but not all observations confirm these sex-related differences in toxicity (97).

Due to the minimal participation of female patients in relevant clinical trials, evaluation of sex differences in cardiotoxicity associated with immune treatment is limited. Female patients may be at higher risk of ICIs-related myocarditis, according to certain research, albeit this has not been proven consistently (98). A study on a pharmacovigilance database seems to identify the female sex (as older age) to be risk factors for ICIs-associated myocarditis but the results could be biased by various confounding factors as the tendency to report unusual or more serious adverse events only and the aforementioned reduced number of women treated for non-small-cell lung cancer representing the principal setting for immunotherapy (99).

Some feelings about a difference between male and female toxicity profile of immunotherapy appear, but a clear conclusion cannot be drawn as a more focused sex and gender-oriented research is needed.

## Older women treatment with anthracyclines

Treatment of old people (age ≥ 65 years) is very challenging, and geriatric patients may be undertreated and exposed to a higher mortality or overtreated and exposed to higher toxicity. Older women are no exception to this rule; furthermore, female patients have their peculiar phenotypes of cardiac disease (already described in the previous chapter) and are underrepresented in clinical trials. Anthracycline cardiotoxicity is dose-related but in the last two decades age has emerged as a relevant risk factor for anthracycline-related HF. Older patients (age > 65 years) showed a greater incidence of HF when compared to younger patients after a cumulative dose of 400 mg/m^2^ (100). In a population of more than 30,000 women with early breast cancer, anthracycline was administered to 18% of patients with the more favorable cardiovascular profile, but still the hazard ratio for cardiomyopathy, HF, and heart disease was 2.48, 1.38, and 1.35, respectively, and this risk was still elevated 5 years after the diagnosis (101). In a population of more than 40,000 patients with breast cancer of which 11% were treated with adjuvant anthracyclines, women aged 66 to 70 years showed an increased risk for HF, whereas women aged 71 to 80 did not (102). Another study of almost 20,000 women documented an increased risk of cardiomyopathy (hazard ratio 1.95), HF, and cardiac dysrhythmias, whereas the association with CAD or conduction disorders was not significantly increased (103).

There are many vulnerabilities linked to older age, and the aging process induces loss of cardiomyocytes, alteration of pharmacokinetics, and the frequent development of comorbidities enhancing chemotherapy-related cardiotoxicity. Among cancer-related risk factors, drug–drug interactions due to the common polypharmacy and lifestyle-dependent risk factors such as physical inactivity and obesity increase the risk of chemotherapy-induced cardiotoxicity, along with a frequent deterioration of renal function as a result of dehydration and/or hypovolemia (104). As far as the sex issue is concerned, the higher risk of anthracycline-induced cardiotoxicity observed in young female patients when compared to young male patients has not been clearly documented in post-menopausal women (105, 106).

Many mechanisms have been proposed to explain enhanced anthracycline-related cardiotoxicity in advanced age. Doxorubicin seems to induce cellular senescence with release of pro-inflammatory cytokines and telomere dysfunction that impairs mitochondrial biogenesis leading to the production of reactive oxygen species (107–109). This effect may be amplified in older female patients, but in the near future this toxic “senescent status” of cells may be targeted and reversed and this fact could reduce the burden of anthracycline-induced cardiotoxicity (110, 111). In last years the immunity system has gained a pivotal role in many diseases, and aging of the immune system (the so-called immunosenescence) has a contributing effect on morbidity and mortality in the elderly (112, 113).

In conclusion, aging of the population will lead to an increasing number of breast cancers in the elderly female patients; these patients are at high risk of cardiotoxicity, but they should not be denied the best treatment. Every effort should be made to reduce the burden of modifiable risk factors and to plan a careful monitoring and follow-up process. This is the point where cardio-oncologists come on stage to help these patients to get their best option care.

## How to manage healthcare sex and gender disparities in cardio-oncology

Disparities related to sex and gender could affect the possibility of female individuals to access to healthcare CO facilities leading to mis- or late diagnosis, un-appropriate early anti-cancer treatment discontinuation, or late referral for cardiovascular toxicity management.

A strong commitment of all CO stakeholders is needed to provide a safe, reliable, and balanced approach to sex and gender issues. First, a sex and gender-focused CO pathway should be available in all CO services. Physicians, nurses, and CO service staff should be warned about the possibility of sex and gender issues and undergo specific training.

Telemedicine could offer the possibility to reduce some of the patients’ concerns about physical, social, racial, and sex and gender issues when referring to a medical facility for a CO consultation. Virtual platforms have proved useful instruments for multidisciplinary discussion and video consultation with staff involved in patient care, with the patient himself or caregiver in family environment (if needed).

Lastly, patient awareness is crucial. All possible efforts shall be made to let patients know that CO programs are familiar with sex- and gender-related issues and that they can find help and tailored solutions into CO services (Figure 1, panel B).

## Conclusion

We are just at the dawning of sex- and gender-related issues in the field of CO. While for anthracyclines and RACD some more robust evidence pointed out the role of sex in predicting side effects of anticancer treatments, for all new drug classes in oncology (in particular immunotherapy) gender-CO is a story to be written. Last but not least, a focused approach on CO social as well as on the quality of life issues of women should be implemented to guarantee a comprehensive care.

## Author contributions

MLC, IB, GG, GR, and AC wrote sections of the manuscript. All authors contributed to the conception of the work, manuscript revision, and approved the submitted version.
